# Decreased serum MG53 levels are associated with SHBG and androgen excess in women with polycystic ovary syndrome

**DOI:** 10.1038/s41598-026-48800-z

**Published:** 2026-04-13

**Authors:** Enes Serhat Coşkun, Fatma Ketenci Gencer, Süleyman Salman, Nilhan Nurlu, Havva Betül Bacak, Serkan Kumbasar, Gözde Yıldırım Timur, Ecenur Çelikoğlu, Şevval Ünlü, Fatma Tunççağ, Rana Güven

**Affiliations:** 1 Department of Obstetrics and Gynecology, Gaziosmanpaşa Training and Research Hospital, Istanbul, Turkey; 2 Department of Biochemistry, Gaziosmanpaşa Training and Research Hospital, Istanbul, Turkey

**Keywords:** Polycystic ovary syndrome, MG53, Mitsugumin-53, SHBG, Androgen excess, Biomarker, Metabolic dysfunction, Insulin resistance, Case–control study, Biomarkers, Diseases, Endocrinology, Medical research

## Abstract

Polycystic ovary syndrome (PCOS) is a common endocrine disorder characterized by hormonal and metabolic abnormalities. Mitsugumin-53 (MG53), a multifunctional E3 ubiquitin ligase, is implicated in insulin signaling and oxidative stress regulation, yet its role in PCOS remains unclear. This study aimed to investigate serum MG53 levels in women with PCOS and explore their associations with hormonal, metabolic, and ovarian parameters. In this case–control study, 64 women with PCOS and 64 healthy controls with comparable age were enrolled. Serum MG53 concentrations were measured by ELISA. Associations between MG53 and continuous variables were assessed using Spearman’s rank correlation. Independent determinants of MG53 were evaluated using multivariable linear regression (including age and BMI as covariates), and diagnostic performance for PCOS was assessed using receiver operating characteristic (ROC) analysis. Serum MG53 concentrations were lower in women with PCOS than in controls (median [IQR]: 124.4 [104.25–202.15] vs. 206.4 [131.63–316.58] pg/mL; *p* = 0.001). MG53 correlated positively with sex hormone–binding globulin (SHBG) (ρ = 0.274, *p* = 0.002) and inversely with ovarian follicle count (ρ =  − 0.223, *p* = 0.011). In multivariable analysis, SHBG remained an independent positive determinant of MG53, whereas hirsutism and follicle count were independent negative predictors. MG53 demonstrated modest discrimination for PCOS (AUC = 0.667, 95% CI 0.569–0.762), with 57.8% sensitivity and 75.0% specificity at an exploratory cut-off of 131.1 pg/mL. Serum MG53 is reduced in PCOS and is associated with SHBG and features related to androgen excess and ovarian morphology. These cross-sectional findings support MG53 as a candidate biomarker reflecting metabolic–reproductive associations in PCOS; however, its diagnostic performance is modest and requires external validation.

*Trial registration* Prospectively registered on ClinicalTrials.gov (NCT07094776) as an observational study.

**Supplementary Information:**

The online version contains supplementary material available at 10.1038/s41598-026-48800-z.

## Introduction

Polycystic ovary syndrome (PCOS) is a common endocrine disorder affecting 6–20% of women of reproductive age and characterized by oligo/anovulation, hyperandrogenism, and polycystic ovarian morphology^1,2^. Beyond its reproductive manifestations, PCOS represents a complex metabolic condition strongly associated with insulin resistance, obesity, dyslipidemia, type 2 diabetes (T2D), and cardiovascular risk^1–4^. Chronic low-grade inflammation, oxidative stress, and stromal fibrosis also contribute to its multifactorial pathophysiology^3,4^. Accordingly, molecules that regulate insulin signaling and cellular stress responses may play pivotal roles in the disorder.

Mitsugumin-53 (MG53, also known as TRIM72) is an E3 ubiquitin ligase originally identified as a skeletal muscle membrane repair protein. Recent studies have expanded its biological relevance to metabolic and cardiometabolic diseases, showing context-dependent effects on insulin sensitivity, lipid metabolism, and inflammatory regulation^5–8^. Elevated circulating MG53 levels have been described in type 2 diabetes and obesity, whereas experimental models of metabolic syndrome have reported decreased MG53 expression associated with impaired tissue repair and mitochondrial stress^6–8^. These bidirectional effects have led to MG53 being characterized as a “double-edged sword” molecule influencing both regenerative and metabolic processes^5,7^.

MG53 modulates several pathways relevant to PCOS pathophysiology, including insulin signaling, PI3K/AKT/mTOR activation, oxidative stress regulation, and inflammatory signaling through NF-κB and inflammasome modulation^9,10^. Furthermore, its roles in mitochondrial homeostasis and fibrosis are of particular interest given that ovarian stromal fibrosis and mitochondrial dysfunction in granulosa cells are key features of PCOS^9,10^. Despite these mechanistic parallels, no prior clinical study has evaluated circulating MG53 levels in women with PCOS.

Given these observations, we hypothesized that MG53 may be involved in the metabolic and ovarian alterations observed in PCOS. Therefore, this study aimed to compare serum MG53 concentrations between women with PCOS and healthy controls, and assess correlations between MG53 levels and hormonal, metabolic, and ovarian parameters. To the best of our knowledge, this is the first clinical study to evaluate circulating MG53 levels in women with PCOS. Elucidating the potential role of MG53 in PCOS may offer new insight into the intersection between reproductive and metabolic dysfunction and identify novel targets for future therapeutic research.

## Methods

### Study design and participants

This prospective, observational case–control study was conducted at the Department of Obstetrics and Gynecology, Gaziosmanpaşa Training and Research Hospital. Participants were consecutively recruited from eligible women who presented to the clinic. Written informed consent was obtained from all participants prior to inclusion in the study. The study was carried out over a three-month period following approval by the Haseki Training and Research Hospital Non-Interventional Clinical Research Ethics Committee (Approval date: June 18, 2025; Decision No: 69–2025). The research was conducted in accordance with the principles of the Declaration of Helsinki and was prospectively registered on ClinicalTrials.gov (NCT07094776) as an observational study.

A total of 128 women were enrolled, including 64 patients with polycystic ovary syndrome (PCOS) and 64 healthy controls with comparable age. PCOS was diagnosed according to the 2003 Rotterdam criteria, as prespecified in the study protocol. PCOM was therefore defined as ≥ 12 follicles measuring 2–9 mm and/or ovarian volume > 10 cm^3^ in at least one ovary. Oligo/anovulation was defined as intermenstrual intervals > 35 days and/or fewer than eight menstrual cycles per year. Clinical hyperandrogenism was assessed using the modified Ferriman–Gallwey (mFG) score, with hirsutism defined as mFG ≥ 8 (or the locally accepted cut-off), and acne evaluated clinically. Biochemical hyperandrogenism was defined as total testosterone above the laboratory-specific upper reference limit. PCOM was defined as ≥ 12 follicles measuring 2–9 mm and/or ovarian volume > 10 cm^3^ in at least one ovary. Ovarian volume was calculated using the prolate ellipsoid formula (0.523 × length × width × thickness).

**Fig. 1:**
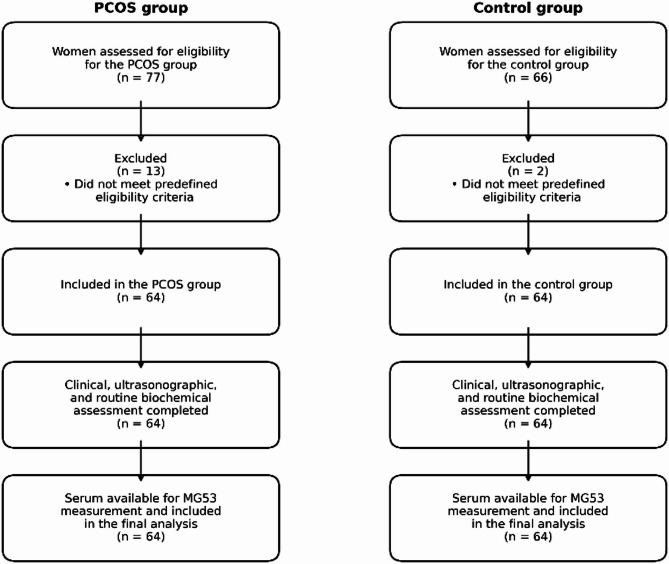
Participant flow diagram screening, exclusions and final inclusion.

Participant selection is shown in Fig. 1. Of 77 women screened for the PCOS group and 66 screened for the control group, 13 and 2 women, respectively, were excluded after application of the predefined eligibility criteria. Clinical, ultrasonographic, and routine biochemical assessments were performed during screening, whereas serum MG53 was measured only in the final included sample of 128 participants.

Before confirming the diagnosis of PCOS, hypothyroidism, hyperthyroidism, hyperprolactinemia, late-onset congenital adrenal hyperplasia, and androgen-secreting tumors were excluded through appropriate biochemical testing.

The control group consisted of women attending the clinic for routine gynecological examination who did not meet the Rotterdam criteria for PCOS, had no sonographic evidence of polycystic ovarian morphology, and had no known endocrine or metabolic disorders. The two groups were comparable in age; BMI was included as a covariate in multivariable analyses.

### Inclusion and exclusion criteria

Women aged 18–45 years who were diagnosed with PCOS according to the Rotterdam criteria and provided written informed consent were included in the study.

Exclusion criteria were as follows: pregnancy or lactation; acute or chronic systemic disease; diabetes mellitus; thyroid dysfunction; hepatic or renal failure; history of malignancy; use of hormonal therapy or metformin within the past six months; presence of chronic inflammatory or autoimmune disorders; use of steroids or medications affecting insulin sensitivity; a body weight change exceeding 10% within the past three months; and engagement in professional athletic activity.

### Clinical and ultrasonographic evaluation

Clinical data including age, height, weight, body mass index (BMI), age at menarche, menstrual cycle pattern, Ferriman–Gallwey score, presence of acne, smoking and alcohol habits, physical activity level, infertility, and history of abortion were recorded for all participants.Regular smoking and alcohol use were defined as consistent consumption within the preceding three months.

All ultrasonographic evaluations were performed by the same experienced investigator using the same ultrasound device (Mindray Resona R9) equipped with a transvaginal probe. Ovarian volume and follicle count were assessed separately for each ovary. To minimize measurement bias, all examinations were conducted by a single operator.

### Blood sampling and storage of serum samples

Venous blood samples were collected in the morning after an overnight fast of at least 8 h, during the early follicular phase (cycle days 2–4) when feasible. In participants with oligo/amenorrhea, sampling was performed either during spontaneous bleeding when available or following progesterone-induced withdrawal bleeding to approximate early follicular phase conditions. After collection, blood was allowed to clot at room temperature and was then centrifuged at 3000 rpm for 10 min to separate the serum. Two serum aliquots were prepared from each participant and stored at –80 °C until analysis. All samples were maintained under single freeze–thaw conditions, and hemolyzed or lipemic specimens were excluded.

### Hormonal and metabolic measurements

Serum levels of follicle-stimulating hormone (FSH), luteinizing hormone (LH), total testosterone, sex hormone-binding globulin (SHBG), anti-Müllerian hormone (AMH), prolactin, thyroid-stimulating hormone (TSH), glucose, insulin, total cholesterol, high-density lipoprotein (HDL), low-density lipoprotein (LDL), triglycerides, C-reactive protein (CRP), aspartate aminotransferase (AST), alanine aminotransferase (ALT), and erythrocyte sedimentation rate were measured using standard laboratory methods.

Hormonal assays were performed using the electrochemiluminescence immunoassay (ECLIA) technique. SHBG was measured in the hospital central laboratory by ECLIA using a Roche cobas e immunoassay analyzer. Insulin resistance was calculated using the homeostasis model assessment (HOMA-IR) formula: HOMA-IR = [fasting glucose (mg/dL) × fasting insulin (µIU/mL)]/405.

### Measurement of MG53 levels

Serum MG53 concentrations were measured using a commercial Human TRIM72 (Mitsugumin-53) ELISA kit (Sinogeneclon Co., Ltd., China; Cat. No: SG-13990). The reported assay sensitivity was 5 pg/mL, with a measurement range of 25–1600 pg/mL, and intra-assay and inter-assay coefficients of variation of < 8% and < 10%, respectively. All samples were analyzed in duplicate, and duplicate measurements with %CV > 10% were re-assayed. A standard curve was generated on each plate using the kit calibrators, and quality control procedures were performed according to the manufacturer’s protocol. Samples with MG53 concentrations exceeding the upper limit of quantification were re-assayed after appropriate dilution following the manufacturer’s instructions. The analyses were performed in a blinded manner by laboratory personnel who were unaware of participants’ group assignments.

### Statistical analysis

Sample size estimation was performed based on an a priori power analysis using a two-tailed Student’s t-test to compare serum MG53 levels between two independent groups. Since no prior data were available on MG53 concentrations in PCOS, a moderate effect size (Cohen’s d = 0.50) was assumed according to Cohen’s convention, with a significance level (α) of 0.05 and power (1 − β) of 0.80. The analysis indicated that a minimum of 64 participants per group (total n = 128) was required to achieve the desired statistical power.

All statistical analyses were performed using IBM SPSS Statistics for Windows, Version 26.0 (IBM Corp., Armonk, NY, USA). Data distribution was assessed with the Shapiro–Wilk test. Normally distributed variables were expressed as mean ± standard deviation (SD), whereas non-normally distributed variables were presented as median (minimum–maximum). Group comparisons were performed using the independent samples t-test for normally distributed variables and the Mann–Whitney U test for non-normally distributed variables. Categorical variables were analyzed with the Chi-square or Fisher’s exact test, as appropriate.

Associations between serum MG53 (TRIM72) levels and clinical, hormonal, and metabolic parameters were evaluated using Spearman’s rank correlation analysis for continuous variables. The predictive ability of MG53 for PCOS was examined through Receiver Operating Characteristic (ROC) curve analysis, and the area under the curve (AUC) with 95% confidence intervals (CI) and p-values were calculated. Given the right-skewed distribution of MG53, sensitivity analyses were performed using log-transformed MG53 values (natural logarithm) and a Gamma generalized linear model with a log link.

A multiple linear regression analysis was performed to identify independent determinants of MG53 concentrations. Variables showing significant correlations with MG53 in univariate analysis were entered into the model as independent predictors. Age and body mass index (BMI) were included as covariates to control for potential confounding, given their established influence on metabolic and hormonal parameters. The regression analysis was performed using the enter method, and model fitness was evaluated with R^2^, adjusted R^2^, and F statistics.

Non-normally distributed variables were analyzed using appropriate non-parametric tests in the primary analyses. No correction for multiple testing was applied, as the analyses were exploratory and intended to identify potential associations for future study rather than to test a prespecified confirmatory hypothesis. Therefore, findings with borderline p-values should be interpreted cautiously. All statistical tests were two-tailed, and a p value < 0.05 was considered statistically significant. There were no missing data. Measurement bias was minimized through a single-center design, the use of standardized procedures, and blinded laboratory analyses.

This study was designed, conducted, and reported in accordance with the *Strengthening the Reporting of Observational Studies in Epidemiology (STROBE)* guidelines. All methodological steps were presented in compliance with the STROBE checklist recommendations.

## Results

### Demographic and clinical characteristics

A total of 128 women were included in the study (64 with PCOS and 64 healthy controls). The groups were comparable in age (24.31 ± 4.94 vs. 24.91 ± 4.97 years, *p* = 0.463), whereas women with PCOS had a significantly higher body mass index (BMI) than controls (26.31 ± 5.00 kg/m^2^ vs. 23.50 ± 4.11 kg/m^2^, *p* = 0.0008). Maximum ovarian volume and bilateral follicle counts were markedly increased in the PCOS group (*p* < 0.001 for all). Menstrual irregularity (81.2% vs. 15.6%), hirsutism (92.2% vs. 46.9%), and acne (50.0% vs. 18.8%) were also more frequent among PCOS patients (all *p* < 0.001). No significant differences were observed in age at menarche, reproductive history, or lifestyle factors such as smoking and alcohol use (all *p* > 0.05) (Table 1).

**Table 1:** Comparison of Demographic and Clinical Characteristics Between PCOS and Control Groups.

Variable	PCOS Median [IQR]/n (%)	Control Median [IQR]/n (%)	*p*-value
Age (years)	23 [20–28]	24 [21–28]	0.463
Body Mass Index (kg/m^2^)	26.3 [22.4–29.1]	23.3 [20.7–25.1]	0.0008
Age at Menarche (years)	13^12–14^	12^12,13^	0.064
Maximum Ovarian Volume (cm^3^)	11.8 [9.0–15.6]	5.4 [4.3–7.1]	< 0.001
Left Ovarian Follicle Count	10.5 [8–12.25]	7^6–8^	< 0.001
Right Ovarian Follicle Count	12^9–12^	7^6–8^	< 0.001
Menstrual Irregularity (Oligomenorrhea/Amenorrhea)	52 (81.2%)	10 (15.6%)	< 0.001
Hirsutism, n (%)	59 (92.2%)	30 (46.9%)	< 0.001
Acne, n (%)	32 (50.0%)	12 (18.8%)	0.0002
History of Pregnancy, n (%)	14 (21.9%)	14 (21.9%)	1.000
History of Miscarriage, n (%)	4 (6.2%)	4 (6.2%)	1.000
History of Infertility, n (%)	8 (12.5%)	6 (9.4%)	0.778
Current Smoking, n (%)	23 (35.9%)	18 (28.1%)	0.449
Alcohol Consumption, n (%)	11 (17.2%)	10 (15.6%)	1.000

Non-parametric variables were analyzed using the Mann–Whitney U test, and categorical variables using Fisher’s Exact test. Data are presented as Median [IQR] or n (%). *P* < 0.05 was considered statistically significant.

### Hormonal and metabolic parameters

Compared with healthy controls, women with PCOS showed significant alterations in hormonal and metabolic parameters (Table 2). Serum TRIM72/MG53 levels were significantly lower in the PCOS group than in controls (124.40 [104.25–202.15] pg/mL vs. 206.40 [131.62–316.57] pg/mL, *p* = 0.001). In contrast, LH, LH/FSH ratio, total testosterone, AMH, fasting glucose, insulin, HOMA-IR, and ALT were significantly higher in PCOS patients (all *p* < 0.05). SHBG (*p* = 0.001) and FSH (*p* = 0.040) levels were lower in the PCOS group. No significant intergroup differences were found in lipid profile (total, HDL, LDL cholesterol, triglycerides), inflammatory markers (CRP, ESR), or thyroid and hepatic enzymes other than ALT (*p* > 0.05 for all).

**Table 2:** Comparison of TRIM72/MG53 and related hormonal and metabolic parameters between PCOS and control groups.

Variable	PCOS(Median [IQR])	Control (Median [IQR])	p
TRIM72/MG53 (pg/mL)	124.40 [104.25–202.15]	206.40 [131.62–316.57]	0.001
LH (IU/L)	6.55 [4.70–9.14]	4.94 [3.73–6.17]	< 0.001
LH/FSH ratio	1.04 [0.80–1.43]	0.67 [0.51–0.95]	< 0.001
Total testosterone (µg/L)	0.32 [0.23–0.38]	0.21 [0.17–0.29]	< 0.001
Sex hormone-binding globulin (SHBG, nmol/L)	58.10 [38.27–70.33]	75.00 [57.70–90.28]	0.001
Anti-Müllerian hormone (AMH, ng/mL)	6.77 [5.64–9.73]	3.65 [1.66–5.02]	< 0.001
HOMA-IR	2.46 [1.87–3.13]	2.04 [1.36–2.50]	0.003
ALT (U/L)	16.00 [13.00–23.00]	14.00 [11.00–19.00]	0.016
Glucose (mg/dL) (mean ± sd)	91.09 ± 7.35	86.56 ± 10.93	0.019
Insulin (mU/L)	11.21 [8.00–14.20]	9.95 [6.18–12.17]	0.030
FSH (IU/L)	6.22 [5.52–7.17]	6.67 [5.71–8.57]	0.040
LDL cholesterol (mg/dL)	102.50 [83.00–125.00]	97.50 [78.75–106.25]	0.080
Erythrocyte sedimentation rate (mm/h)	7.00 [4.00–12.25]	6.00 [2.00–9.00]	0.122
AST (U/L)	17.00 [15.00–19.00]	15.00 [14.00–19.00]	0.152
Total cholesterol (mg/dL)	179.50 [148.75–207.50]	173.00 [152.75–196.00]	0.298
HDL cholesterol (mg/dL)	55.50 [47.75–62.25]	56.50 [48.00–65.25]	0.418
C-reactive protein (CRP, mg/L)	4.00 [1.00–8.00]	3.00 [1.00–5.33]	0.433
Thyroid-stimulating hormone (TSH, mIU/L)	1.35 [1.16–1.96]	1.48 [1.18–2.09]	0.457
Prolactin (ng/mL)	11.29 [8.19–15.44]	10.50 [8.22–14.07]	0.740
Triglycerides (mg/dL)	93.50 [66.25–121.75]	88.00 [60.50–131.25]	0.782

Data are presented as mean ± SD for normally distributed variables and as median [IQR] for non-normally distributed variables. Between-group comparisons were performed using Welch’s t-test for normally distributed variables and the Mann–Whitney U test for non-normally distributed variables. *P* < 0.05 was considered statistically significant.

### Correlations of serum MG53 with clinical and biochemical parameters

Spearman’s rank correlation was used to assess associations between circulating MG53 (TRIM72) levels and continuous clinical/biochemical variables (Table 3). MG53 correlated positively with HDL cholesterol (ρ = 0.299, *p* < 0.001) and sex hormone–binding globulin (SHBG) (ρ = 0.274, *p* = 0.002). Inverse correlations were observed with C-reactive protein (CRP) (ρ =  − 0.197, *p* = 0.026), maximum ovarian volume (ρ =  − 0.239, *p* = 0.007), and ovarian follicle count (ρ =  − 0.223, *p* = 0.012).

**Table 3:** Significant Spearman correlations between circulating MG53 (TRIM72) levels and clinical, hormonal, and metabolic parameters.

Parameter	Spearman’s ρ	*p*-value
HDL cholesterol	0.299	< 0.001
Sex hormone-binding globulin	0.274	0.002
C-reactive protein	− 0.197	0.026
Maximum Ovarian Volume	− 0.239	0.007
Ovarian follicle count	− 0.223	0.012

Data are presented as Spearman’s correlation coefficients (ρ) and corresponding *p*-values. Correlations were analyzed using Spearman’s rank test. Only statistically significant associations (*p* < 0.05) are displayed.

### Multiple linear regression analysis

A multiple linear regression model was constructed to identify independent determinants of serum MG53 levels, adjusting for age and BMI as potential confounders (Table 4). The overall model was significant (*F*(9,118) = 3.10, *p* = 0.003), explaining 18.2% of the variance in MG53 concentrations (adjusted *R*^*2*^ = 0.123). SHBG was an independent positive predictor of MG53 (β = 2.52, *p* = 0.010), whereas hirsutism (β =  − 186.53, *p* = 0.018) and ovarian follicle count (β =  − 34.10, *p* = 0.047) were independent negative predictors. No other variables, including HDL cholesterol, CRP, PCOS status, ovarian volume, age, or BMI, remained significant after adjustment.

**Table 4:** Multiple linear regression analysis showing independent predictors of circulating MG53 (TRIM72) levels.

Variable	B	SE	*t*	*p*-value	95% CI
HDL cholesterol	0.48	2.32	0.21	0.836	− 4.11 – 5.07
Sex hormone-binding globulin (SHBG)	2.52	0.96	2.63	0.010	0.62 – 4.42
C-reactive protein	− 3.62	5.52	− 0.66	0.513	− 14.56 – 7.31
Hirsutism	− 186.53	77.98	− 2.39	0.018	− 340.94 – − 32.11
PCOS	80.32	96.30	0.83	0.406	− 110.37 – 271.02
Maximum Ovarian Volume	3.13	8.95	0.35	0.727	− 14.59 – 20.85
Ovarian Follicle Count	* − 34.10*	*16.99*	* − 2.01*	*0.047*	* − 67.74 – − 0.46*
Age	9.09	6.27	1.45	0.150	− 3.32 – 21.50
Body Mass Index	10.82	7.35	1.47	0.143	− 3.73 – 25.37

Model statistics: n = 128, R^2^ = 0.182, adjusted R^2^ = 0.123, F(9,118) = 3.10, p(model) = 0.003. Regression coefficients (B) are presented with standard errors and 95% confidence intervals.

Age and BMI were included as covariates to control for potential demographic and anthropometric confounding.

Sensitivity analyses addressing the right-skewed distribution of MG53 showed broadly consistent directions of association. In models using log-transformed MG53, hirsutism and follicle count remained inversely associated with MG53, whereas the positive association with SHBG was attenuated to borderline significance. A Gamma generalized linear model with a log link yielded similar directional findings and supported an independent positive association between SHBG and MG53 (Supplementary Table S1).

### Receiver operating characteristic (ROC) analysis

**Fig. 2:**
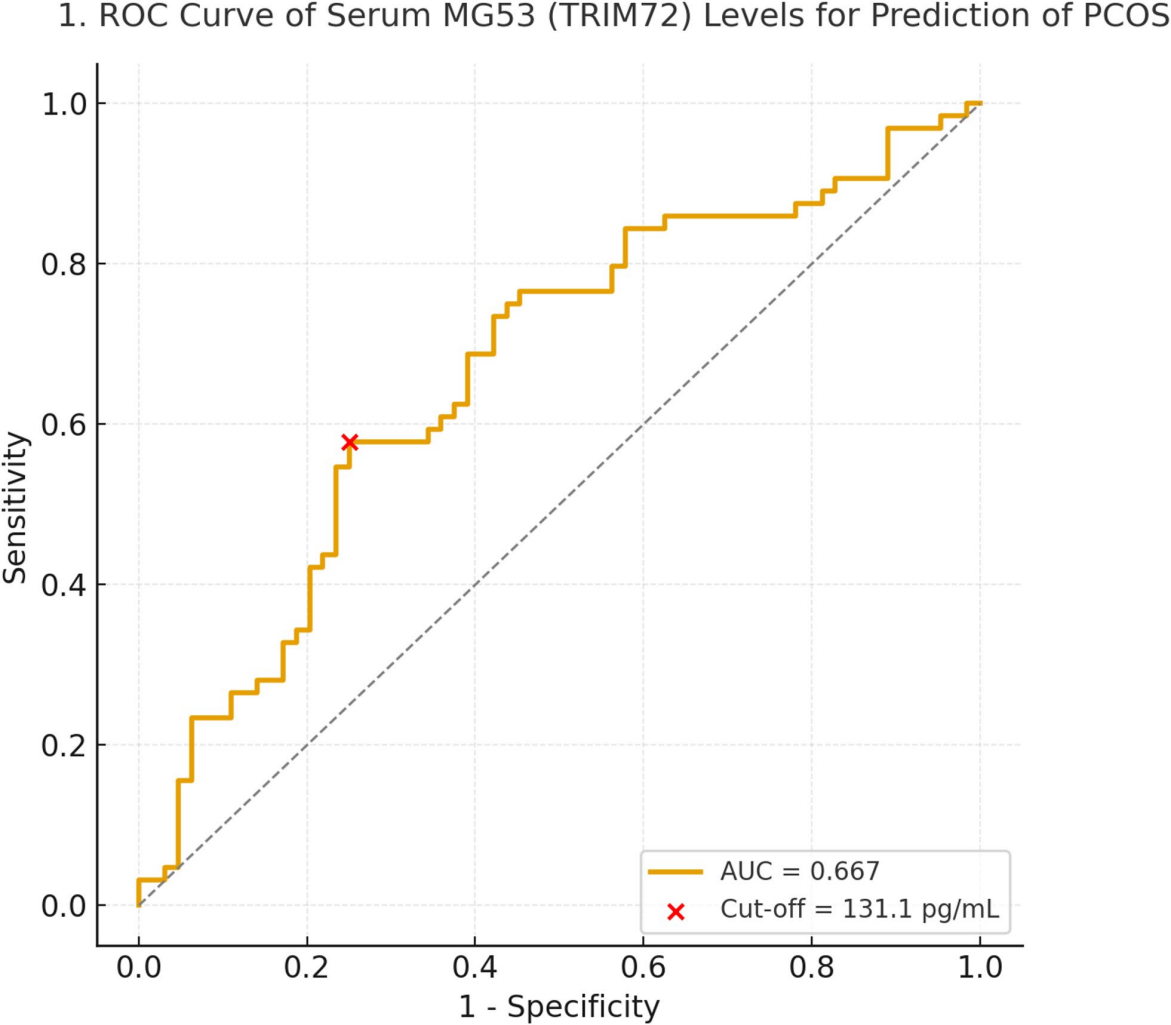
ROC Curve of Serum MG53 (TRIM72) Levels for Prediction of PCOS.

**Fig. 3:**
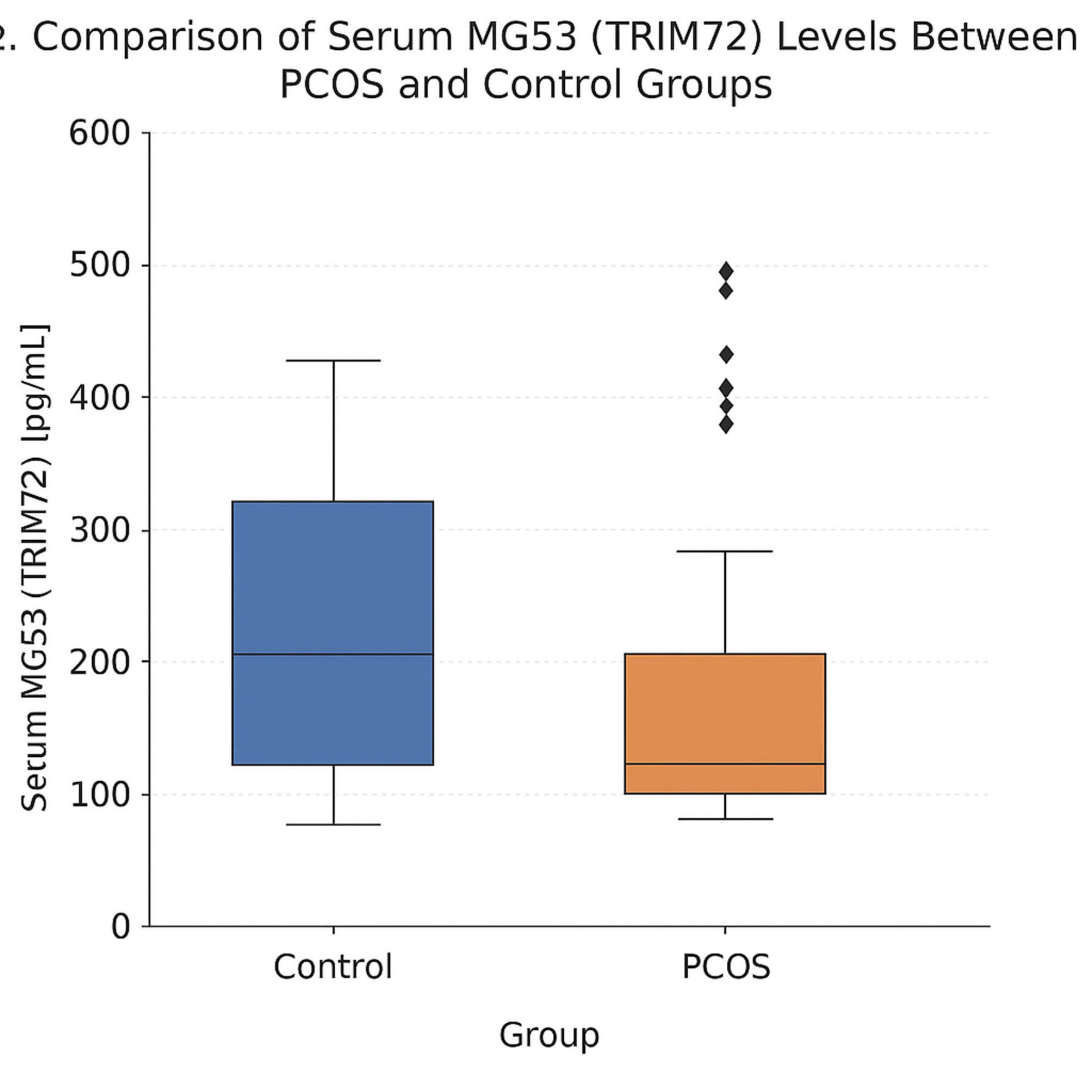
Comparison of Serum MG53 (TRIM72)Levels Between PCOS and Control Groups.

ROC curve analysis showed modest discrimination for PCOS (AUC = 0.667, 95% CI 0.569–0.762). The proposed cut-off of 131.1 pg/mL should be considered exploratory, as it was derived from the current dataset and has not been externally validated. (Figs. 2, 3).

## Discussion

This study presents the first clinical evidence demonstrating that serum Mitsugumin-53 (MG53, also known as TRIM72) levels are significantly decreased in women with polycystic ovary syndrome (PCOS). Moreover, MG53 levels were closely associated with both metabolic and ovarian parameters. MG53 showed a positive correlation with sex hormone-binding globulin (SHBG) and HDL cholesterol, and a negative correlation with hirsutism score, follicle count, and ovarian volume. These findings suggest that MG53, an E3 ubiquitin ligase involved in membrane repair and metabolic regulation, may play a previously unrecognized role in the pathophysiology of PCOS.

MG53 has been mainly studied in the context of metabolic and cardiometabolic disorders^5–8^. Increased MG53 levels have been reported in type 2 diabetes and obesity, where MG53 impairs insulin signaling by promoting ubiquitination and degradation of the insulin receptor and IRS-1, and acts as a glucose-sensitive myokine that regulates systemic insulin response^5,6^. Conversely, decreased MG53 levels have been observed in metabolic syndrome and tissue injury models, which are associated with reduced membrane repair capacity and enhanced oxidative stress^8,11,12^. The reduction of MG53 in PCOS may reflect a complex metabolic–inflammatory phenotype characterized by insulin resistance, chronic inflammation, mitochondrial dysfunction, and fibrotic remodeling^3,4^. These findings imply that MG53 may be involved not only in glucose metabolism but also in inflammatory and regenerative processes relevant to PCOS.

MG53 has been implicated in the regulation of endoplasmic reticulum (ER) stress and tissue remodeling.In diabetic wound models, suppression of TRIM72 alleviated ER stress and enhanced repair capacity, indicating that excessive MG53 activation may impair regeneration in certain pathological contexts^13^.Furthermore, MG53 regulates fibrosis and cellular proliferation through the PI3K/AKT/mTOR signaling pathway.Experimental evidence shows that MG53 overexpression enhances hepatic stellate cell activation and extracellular matrix deposition, supporting its profibrotic role in liver injury models.Consistently, PCOS ovaries exhibit increased TGF-β expression and collagen accumulation, reflecting fibrotic remodeling of the ovarian stroma^3,4^.Thus, dysregulated MG53 signaling may influence ovarian extracellular matrix dynamics in a similar manner. MG53 can also modulate inflammatory responses by interacting with the NF-κB and inflammasome signaling pathways. In infectious models, MG53 overexpression was shown to suppress host immunity against Candida albicans by inhibiting NF-κB activation and inflammasome assembly^14^. Beyond its protein-level effects, MG53 expression is also regulated epitranscriptomically through m6A RNA methylation mediated by YTHDF2, which influences oxidative stress and inflammatory responses^15^. In the context of PCOS, reduced MG53 levels may therefore contribute to the heightened inflammatory and oxidative milieu characteristic of the disorder^4^. Collectively, these multifaceted and context-dependent actions illustrate the dual nature of MG53, which can exert either protective or detrimental effects depending on cellular and metabolic conditions. This context-specific behavior has led to its characterization as a “double-edged sword” in the literature^16^.

Mitochondrial dysfunction in granulosa cells has been implicated in impaired folliculogenesis and reduced oocyte quality^3^.Given that MG53 supports mitochondrial integrity and energy homeostasis^11,12,15,17^, lower MG53 levels may contribute to cellular stress and disrupted follicular development in PCOS.Recent evidence also indicates that MG53 can activate the Nrf2/HO-1 antioxidant pathway through its interaction with Annexin A1^17^ and enhance mitochondrial repair in neuronal models^12^, further supporting its role in maintaining cellular homeostasis.The negative correlations between MG53 and both follicle count and ovarian volume in the present study support this hypothesis. No significant correlations were observed between MG53 and AMH or gonadotropins, suggesting that MG53 may be more closely related to stromal metabolic activity than to ovarian reserve per se.

The positive association between MG53 and SHBG highlights the protein’s link to metabolic and hormonal balance. Low SHBG levels are closely associated with hyperandrogenism and insulin resistance^3,4^. Parallel changes in MG53 and SHBG may therefore reflect shared regulatory mechanisms involving hepatic function and inflammatory status. The positive correlation with HDL cholesterol and the negative correlation with CRP further support MG53’s role in maintaining metabolic–inflammatory homeostasis^7,8,14^.

The observed positive association between MG53 and SHBG may represent a key metabolic–hormonal interface in PCOS. SHBG is synthesized primarily in the liver and binds circulating androgens, limiting their bioavailability. Reduced SHBG concentrations lead to higher levels of free testosterone, which intensifies clinical hyperandrogenism, including hirsutism and acne^3,4^. MG53, through its regulatory effects on hepatic insulin signaling and the PI3K/AKT/mTOR pathway^18^, may indirectly influence SHBG synthesis. In insulin-resistant states, elevated insulin suppresses hepatic SHBG production; therefore, decreased MG53 expression could potentiate this suppression by impairing insulin sensitivity. Conversely, adequate MG53 activity may support hepatic metabolic stability and favor SHBG synthesis, thereby mitigating androgen excess.

Additionally, MG53’s modulation of inflammatory and oxidative stress pathways via NF-κB and Nrf2 signaling^10,14,15,17^ could further affect hepatic transcriptional control of SHBG. These interactions suggest that MG53 deficiency may act as a permissive factor for androgen-driven manifestations in PCOS through a combined effect on SHBG production, insulin resistance, and inflammatory tone. The negative correlation between MG53 and hirsutism found in this study supports this mechanistic link. Collectively, these findings point toward MG53 as a potential upstream regulator of the hepatic–endocrine crosstalk governing androgen bioavailability in PCOS.

In multiple linear regression analysis, SHBG remained an independent positive determinant of MG53, whereas hirsutism and follicle count were independent negative predictors. Age and body mass index (BMI) were included as covariates because both are potential confounding factors known to influence metabolic markers. This approach enabled a clearer assessment of the independent relationships between hormonal, metabolic, and morphological parameters and MG53. The association between MG53 and SHBG was directionally consistent across sensitivity analyses, although some attenuation after log transformation suggests that this finding may be somewhat sensitive to model specification and should be interpreted with caution pending replication. Receiver operating characteristic (ROC) analysis revealed that MG53 had a modest diagnostic accuracy for predicting PCOS (AUC = 0.667, 95% CI 0.569–0.762; sensitivity 57.8%, specificity 75.0). These findings suggest that MG53 alone has limited diagnostic value but may serve as an adjunct biomarker when combined with hormonal and metabolic parameters^7^. In future research, an integrated predictive model incorporating MG53 together with hormonal and metabolic indicators could be developed to improve diagnostic accuracy for PCOS. Such an approach may represent a promising direction for subsequent studies.

Given the exploratory design and the number of comparisons performed, these associations should be interpreted with caution, especially those with borderline statistical significance, and require confirmation in larger independent cohorts.

## Limitations and strengths

While our findings support an association between circulating MG53 and hormonal/metabolic parameters as well as ovarian morphology in PCOS, several limitations should be considered. First, the cross-sectional design precludes causal inference and does not establish directionality. Second, this single-center study with a modest sample size may be susceptible to residual confounding, limits power for detailed subgroup analyses, and may restrict the generalizability of the findings. Third, BMI differed between groups; although we adjusted for age and BMI in multivariable models, confounding related to adiposity and lifestyle factors cannot be fully excluded. In addition, the modest sample size limited the robustness of normal-weight subgroup analyses or matching-based approaches, and these strategies should be addressed in larger future studies. Fourth, MG53 was measured at a single time point and tissue-level expression was not assessed; therefore, mechanistic interpretation remains hypothesis-generating. Finally, multiple associations were explored without formal correction for multiple testing, which increases the risk of type I error. Accordingly, statistically significant findings—particularly those with borderline p-values—should be interpreted cautiously and considered hypothesis-generating until replicated in independent cohorts. In addition, polycystic ovarian morphology was defined according to the 2003 Rotterdam criteria prespecified in the study protocol. More recent international guidelines recommend higher follicle number thresholds when contemporary ultrasound technology is used, which may limit direct comparability with newer studies. The strengths of this study include standardized sampling during the follicular phase, well-defined clinical and ultrasound characterization of participants, and blinded laboratory measurements.

## Clinical implications

The ROC analysis indicated modest discrimination (AUC ≈ 0.67), suggesting that MG53 alone is unlikely to be clinically useful as a stand-alone diagnostic marker. Accordingly, the proposed cut-off should be considered exploratory and requires external validation before clinical application. Future studies should assess whether MG53 adds incremental value to established clinical, hormonal, and metabolic markers within multivariable prediction models and whether MG53 relates to clinically meaningful outcomes such as metabolic risk stratification or treatment response.

## Conclusions

In this case–control study, serum MG53 levels were lower in women with PCOS than in controls and were associated with SHBG and features related to androgen excess and ovarian morphology. These findings support MG53 as a candidate biomarker reflecting metabolic–reproductive associations in PCOS; however, its diagnostic performance was modest (AUC = 0.667), and the proposed cut-off (131 pg/mL) should be regarded as exploratory. Larger, multi-center studies with external validation and mechanistic investigations (including tissue-level expression) are warranted to clarify the biological role and potential clinical utility of MG53 in PCOS.

## Supplementary Information

Below is the link to the electronic supplementary material.

Supplementary Material 1

Supplementary Material 2

## Data Availability

The dataset supporting the conclusions of this article is included as an additional file in Excel format (dataset.xlsx).

## Abbreviations

AUC: Area under the curve
AMH: Anti-Müllerian hormone
ALT: Alanine aminotransferase
AST: Aspartate aminotransferase
BMI: Body mass index
CRP: C-reactive protein
ECLIA: Electrochemiluminescence immunoassay
ER: Endoplasmic reticulum
FSH: Follicle-stimulating hormone
HDL: High-density lipoprotein
HOMA-IR: Homeostasis model assessment of insulin resistance
IRS-1: Insulin receptor substrate-1
LDL: Low-density lipoprotein
LH: Luteinizing hormone
MG53: Mitsugumin-53
mTOR: Mammalian target of rapamycin
NF-κB: Nuclear factor kappa-light-chain-enhancer of activated B cells
PCOS: Polycystic ovary syndrome
ROC: Receiver operating characteristic
SD: Standard deviation
SE: Standard error
SHBG: Sex hormone-binding globulin
STROBE: Strengthening the Reporting of Observational Studies in Epidemiology
TG: Triglyceride
TGF-β: Transforming growth factor beta
TSH: Thyroid-stimulating hormone
WHR: Waist-to-hip ratio
