# Supplementary material for: Decreased serum MG53 levels are associated with SHBG and androgen excess in women with polycystic ovary syndrome
Source: Sci Rep. 2026 Apr 13;16:15753. doi: 10.1038/s41598-026-48800-z (PMC13190848; doi:10.1038/s41598-026-48800-z)
Supplement: Supplementary file 2 — Supplementary Material 2 [file 41598_2026_48800_MOESM2_ESM.docx]

Supplementary Table S1. Sensitivity analyses addressing the right-skewed distribution of MG53

Models include HDL, SHBG, CRP, hirsutism (0/1), PCOS status (0/1), maximum ovarian volume, left ovarian follicle count, age, and BMI (n = 128).

A) Multivariable linear regression with log-transformed MG53 (ln[MG53]) as the dependent variable

| Predictor | B | SE | p value | 95% CI (lower) | 95% CI (upper) |
| --- | --- | --- | --- | --- | --- |
| HDL (mg/dL) | 0.007 | 0.005 | 0.131 | -0.002 | 0.016 |
| SHBG (nmol/L) | 0.003 | 0.002 | 0.067 | 0.000 | 0.007 |
| CRP (mg/L) | -0.011 | 0.011 | 0.306 | -0.032 | 0.010 |
| Hirsutism (0/1) | -0.608 | 0.152 | <0.001 | -0.909 | -0.308 |
| PCOS status (0/1) | 0.176 | 0.187 | 0.348 | -0.195 | 0.547 |
| Maximum ovarian volume (cm³) | -0.004 | 0.017 | 0.812 | -0.039 | 0.030 |
| Left ovarian follicle count | -0.066 | 0.033 | 0.048 | -0.132 | -0.001 |
| Age (years) | 0.019 | 0.012 | 0.118 | -0.005 | 0.043 |
| BMI (kg/m²) | 0.028 | 0.014 | 0.053 | 0.000 | 0.056 |

B) Gamma generalized linear model (log link) with MG53 as the dependent variable

| Predictor | Coefficient | SE | p value | 95% CI (lower) | 95% CI (upper) |
| --- | --- | --- | --- | --- | --- |
| HDL (mg/dL) | 0.007 | 0.007 | 0.264 | -0.006 | 0.020 |
| SHBG (nmol/L) | 0.007 | 0.003 | 0.016 | 0.001 | 0.012 |
| CRP (mg/L) | -0.021 | 0.016 | 0.173 | -0.052 | 0.009 |
| Hirsutism (0/1) | -0.521 | 0.221 | 0.019 | -0.955 | -0.087 |
| PCOS status (0/1) | 0.191 | 0.273 | 0.485 | -0.345 | 0.727 |
| Maximum ovarian volume (cm³) | 0.002 | 0.025 | 0.926 | -0.047 | 0.052 |
| Left ovarian follicle count | -0.111 | 0.048 | 0.022 | -0.205 | -0.016 |
| Age (years) | 0.032 | 0.018 | 0.071 | -0.003 | 0.067 |
| BMI (kg/m²) | 0.032 | 0.021 | 0.123 | -0.009 | 0.073 |

Abbreviations: BMI, body mass index; CRP, C-reactive protein; HDL, high-density lipoprotein; SHBG, sex hormone-binding globulin.
